# Supplementary material for: Impact of diabetes status on immunogenicity of trivalent inactivated influenza vaccine in older adults
Source: Influenza Other Respir Viruses. 2021 Dec 3;16(3):562–7. doi: 10.1111/irv.12933 (PMC8983908; doi:10.1111/irv.12933)
Supplement: Supplementary file 1 — Table S1. Descriptive characteristics of diabetic and non‐diabetic participants Table S2. Frequency of responses to functional status measures among diabetic and non‐diabetic adult participants. Table S3. Predictors of rate of change between D21 and D365 Table S4. Pre‐ and post‐vaccination frequency of antibody‐secreting cells (ASCs) to influenza vaccine reference antigens among participants aged 50–80 years with and without type 2 diabetes mellitus. [file IRV-16-562-s001.docx]

**Supplemental Table 1. Descriptive characteristics of diabetic and non-diabetic participants**

|  | **Diabetic (N=92)** | | **Non-Diabetic (N=113)** | | **p-value** |
| --- | --- | --- | --- | --- | --- |
|  | **N (%) or mean (sd)** | | **N (%) or mean (sd)** | |  |
| **Age, Mean** | 65.6 | (7.7) | 61.9 | (7.4) | 0.001 |
| **Sex** |  |  |  |  | 0.03 |
| Male | 49 | (53) | 43 | (38) |  |
| Female | 43 | (47) | 70 | (62) |  |
| **Site** |  |  |  |  | 0.4 |
| Wisconsin | 70 | (76) | 80 | (71) |  |
| Pennsylvania | 22 | (24) | 33 | (29) |  |
| **Race/Ethnicity** |  |  |  |  | 0.7 |
| White, non-Hispanic | 78 | (85) | 100 | (89) |  |
| Black, non-Hispanic | 13 | (14) | 11 | (10) |  |
| Other | 1 | (1) | 2 | (2) |  |
| **Body Mass Index (kg/m^2^)** | 35.2 | (7.4) | 30.3 | (6.5) | <0.001 |
| **Obese (Body Mass Index ≥30 kg/m^2^)** | 69 | (75) | 55 | (49) | <0.001 |
| **HbA1c^1^ (n=88)** | 7.3 | (1.3) | NA |  | NA |
| **Vitamin D level (ng/mL)** | 43.8 | (17.0) | 41.2 | (16.9) | 0.28 |
| **Vitamin D <30 ng/mL** | 17 | (18.5) | 25 | (22.1) | 0.52 |
| **Smoked >100 cigarettes in lifetime** | 51 | (55) | 49 | (44) | 0.1 |
| **Current health assessment^2^**  **Scale 0 (worst) – 100 (best)** | 68.7 | (17.3) | 81.4 | (13.3) | <0.001 |

^1^ Serum HbA1c concentration was measured for 88 participants with diabetes.

^2^ The validated EQ-5D health ruler [20] was used to assess participant health on the day of enrollment. Data are presented on a scale of 1 (worst) to 100 (best).

**Supplemental Table 2. Frequency of responses to functional status measures among diabetic and non-diabetic adult participants.**

|  | **Diabetic (N=92)** | **Non-Diabetic (N=113)** | **p value** |
| --- | --- | --- | --- |
|  | **N (%)** | **N (%)** |  |
| **Self-reported mobility** |  |  | 0.5 |
| No problems walking about | 47 (51) | 66 (58) |  |
| Some problems walking about or confined to bed | 45 (49) | 47 (42) |  |
| **Self-rated ability to perform self-care** |  |  | 0.7 |
| No problems | 65 (71) | 80 (71) |  |
| Some problems washing/dressing or unable to  wash/dress self | 27 (29) | 33 (29) |  |
| **Usual activities** |  |  | 0.4 |
| No problems performing usual activities | 53 (58) | 75 (66) |  |
| Some problems performing usual activities or  unable to perform usual activities | 38 (42) | 38 (34) |  |
| **Pain** |  |  | 0.1 |
| I have no pain or discomfort | 30 (33) | 53 (47) |  |
| Moderate or extreme pain or discomfort | 62 (67) | 60 (53) |  |
| **Anxiety** |  |  | 0.8 |
| Not anxious/depressed | 60 (65) | 77 (68) |  |
| Moderately/extremely depressed | 32 (35) | 36 (32) |  |

**Supplemental Table 3. Predictors of rate of change between D21 and D365**

|  | **A/CA/07/09(H1N1)pdm09** | | **A/Victoria/361/11(H3N2)** | | **B/Brisbane/60/08 (Vic)** | | **B/Wisconsin/1/10(Yam)** | |
| --- | --- | --- | --- | --- | --- | --- | --- | --- |
|  | Coefficient (SE) | p-value | Coefficient (SE) | p-value | Coefficient (SE) | p-value | Coefficient (SE) | p-value |
| Age | -0.02 (0.01) | 0.18 | -0.02 (0.01) | 0.18 | 0.02 (0.01) | 0.05 | -0.01 (0.01) | 0.67 |
| Diabetes | -0.25 (0.20) | 0.23 | -0.15 (0.18) | 0.43 | -0.04 (0.16) | 0.79 | -0.001 (0.23) | 0.99 |
| White race | 0.29 (0.30) | 0.33 | -0.41 (0.28) | 0.14 | -0.04 (0.23) | 0.87 | 0.29 (0.31) | 0.36 |
| Sex | 0.01 (0.19) | 0.98 | -0.22 (0.17) | 0.21 | -0.14 (0.15) | 0.35 | -0.06 (0.21) | 0.78 |
| Body mass index | 0.02 (0.01) | 0.14 | -0.02 (0.01) | 0.23 | -0.01 (0.01) | 0.41 | -0.02 (0.02) | 0.26 |
| Impaired functional status^1^ | -0.39 (0.20) | 0.06 | -0.05 (0.18) | 0.77 | -0.29 (0.16) | 0.08 | -0.12 (0.24) | 0.63 |
| Vitamin D level | 0.32 (0.24) | 0.20 | 0.23 (0.22) | 0.30 | 0.15 (0.19) | 0.42 | 0.37 (0.27) | 0.17 |
| D21 HI titer | -0.27 (0.06) | <0.01 | -0.27 (0.05) | <0.01 | -0.36 (0.05) | <0.01 | -0.38 (0.06) | <0.01 |

Note: Separate linear regression models were estimated for HI titers to each reference virus.

^1^ A participant was considered to have impaired functional status if he/she indicated any problems with mobility, ability to perform self-care, usual activities, pain, or anxiety.

**Supplemental Table 4. Pre- and post-vaccination frequency of antibody-secreting cells (ASCs) to influenza vaccine reference antigens among participants aged 50-80 years with and without type 2 diabetes mellitus.**

|  | **Diabetic (N=9)** | | | | **Non-Diabetic (N=14)** | | |  |
| --- | --- | --- | --- | --- | --- | --- | --- | --- |
|  | GMP (CIs) | | GMP ratio  (D21/D0) | | GMP (CIs) | | GMP ratio (D21/D0) | P value^1^ |
|  | D0 | D21 |  |  | D0 | D21 |  |  |
| **A/CA/07/09 (H1N1)pdm09** | | | | | | | |  |
| IgG | 0.4 (0.12,1.36) | 3.58 (1.93, 6.62) | | 8.88 (2.66, 29.65) | 0.81 (0.33, 2.03) | 4.36 (2.42, 7.86) | 5.37 (1.92, 15.06) | NS |
| IgM | 2.2 (0.38, 12.74) | 10.04 (4.39, 22.98) | | 4.57 (1.22, 17.09) | 7.36 (2.81, 19.28) | 7.32 (3.92, 13.69) | 0.99 (0.59, 1.68) | 0.01 |
| IgA | 0.05 (0.01, 0.18) | 0.5 (0.12, 2.03) | | 10.37 (1.62, 66.42) | 0.21 (0.07, 0.62) | 0.77 (0.22, 2.72) | 3.73 (0.72, 19.29) | NS |
| **A/Victoria/210/09 (H3N2)** | | | | | | | |  |
| IgG | 0.12 (0.03, 0.55) | 2.43 (1.07, 5.54) | | 20.31 (2.54, 162.17) | 0.29 (0.1, 0.86) | 1.92 (0.49, 7.5) | 6.66 (2.32, 19.1) | NS |
| IgM | 9.38 (3.35, 26.32) | 10.43 (4.64, 23.45) | | 1.11 (0.44, 2.8) | 9.68 (3.42, 27.4) | 9.91 (4.51, 21.8) | 1.02 (0.54, 1.93) | NS |
| IgA | 0.28 (0.05, 1.63) | 0.57 (0.15, 2.18) | | 2.07 (0.23, 18.26) | 0.28 (0.09, 0.87) | 0.43 (0.09, 1.95) | 1.54 (0.64, 3.72) | NS |
| **B/Brisbane/60/2008 (Victoria)** | | | | | | | |  |
| IgG | 0.2 (0.05, 0.81) | 2.81 (1.52, 5.2) | | 14.17 (2.92, 68.81) | 0.82 (0.34, 1.96) | 2.88 (1.04, 7.96) | 3.5 (1.16, 10.59) | NS |
| IgM | 0.78 (3.3, 18.66) | 8.65 (4.26, 17.53) | | 1.1 (0.62, 1.95) | 7.26 (3.08, 17.15) | 9 (3.71, 21.79) | 1.24 (0.54, 2.82) | NS |
| IgA | 0.26 (0.04, 1.83) | 1.38 (0.26, 7.33) | | 5.33 (0.76, 37.55) | 0.69 (0.31, 1.55) | 1.35 (0.51, 3.57) | 1.96 (0.86, 4.47) | NS |

GMP=geometric mean percentage; NS=non-significant (p>0.05).

^1^ Comparison of GMP ratio (D21/D0) between diabetic and non-diabetic groups.
